# Assessment of Patellar Lateralization and Its Association with Patellofemoral Osteoarthritis Severity: A Retrospective MRI-Based Study

**DOI:** 10.3390/diagnostics16182997

**Published:** 2026-09-16

**Authors:** Do Yeon Ahn, Hee Jin Park, Ji Na Kim, Myung Sub Kim

**Affiliations:** Department of Radiology, Kangbuk Samsung Hospital, Sungkyunkwan University School of Medicine, #108 Pyung-dong, Jongno-gu, Seoul 03181, Republic of Korea; cat3305@nate.com (D.Y.A.); hebecrom@hanmail.net (J.N.K.); smmgkim84@naver.com (M.S.K.)

**Keywords:** magnetic resonance imaging, patellofemoral joint, osteoarthritis, patellar lateralization, patellar instability, patellofemoral alignment

## Abstract

**Background/Objectives:** Alteration of the anatomy or congruence of the patellofemoral joint may lead to osteoarthritis. The distance of patellar lateralization in a coronal superimposed MR image (coronal MRI superimposition technique) can be a useful method for evaluating patellar lateralization. **Methods:** We correlated the presence of patellofemoral osteoarthritis (PFOA) with the tibial tuberosity–trochlear groove (TT-TG) distance and the distance of patellar lateralization. We also evaluated the correlation between the presence of patellar lateralization and the severity of PFOA. This retrospective study included 202 patients (non-severe PFOA, *n* = 135; severe PFOA, *n* = 67) who underwent knee magnetic resonance imaging (MRI). PFOA criteria and severity were defined using the International Cartilage Repair Society (ICRS) classification system. All MRI images were independently evaluated by two musculoskeletal radiologists. After dividing the study group into a patellar lateralization group and a control group according to the distance between the patellar long axis and the deepest portion of the trochlear deep notch with a 7 mm threshold, the association between patellar lateralization and PFOA severity was analyzed. **Results:** Interobserver agreement was excellent for measuring TT-TG distance (ICC = 0.980) and patellar lateralization (ICC = 0.984). The mean distance of patellar lateralization was significantly greater in the severe PFOA group (ICRS grades 2–4) compared to the non-severe group (ICRS grades 0–1) (6.02 mm vs. 4.78 mm; 95% CI of the difference, −2.110 to −0.376; *p* = 0.005). Also, we found that as the International Cartilage Repair Society (ICRS) grade of the PFOA rose, the proportion of patellar lateralization increased more than normal in each grade (*p* = 0.009). There were statistically significant differences in the TT-TG distance and patellar lateralization between the non-severe group (Grade 0 and Grade 1) and the severe PFOA group (more than Grade 2) by both interpreters (*p* = 0.029 and 0.014 for TT-TG distance; *p* = 0.005 and 0.002 for patellar lateralization). **Conclusions:** The measurement of patellar lateralization using the coronal MRI superimposition technique on MRI is significantly associated with PFOA and its severity. Thus, the coronal MRI superimposition technique would be a useful method for evaluating the relationship between patellar lateralization and PFOA.

## 1. Introduction

Osteoarthritis (OA) is a major global public health challenge and one of the leading causes of musculoskeletal disability worldwide [1,2]. In 2020, 595 million people worldwide suffered from OA, a number which is predicted to increase by 74.9% by 2050. Currently, OA is recognized not simply as a process of isolated cartilage wear, but as a complex, heterogeneous disease of the entire joint. The pathogenesis of OA encompasses cartilage degradation as well as subchondral bone remodeling, fibrosis and inflammation of the synovial membrane, degenerative changes to the ligaments and menisci, and pathological alterations to periarticular tissues such as the subchondral fat pad. The development of this disease is driven by a combination of established risk factors, including advanced age, female gender, a high body mass index, abnormal joint loading, and potential systemic factors such as trace element imbalances. Mechanistically, a disruption of joint homeostasis shifts the environment toward catabolic activity, leading to extracellular matrix degradation, increased oxidative stress, and the release of pro-inflammatory cytokines that perpetuate structural damage across all joint tissues [3,4].

The two most common types of knee OA are combined femorotibial OA and patellofemoral OA, followed by isolated patellofemoral OA (PFOA) [5]. Approximately 9% of the population over 40 years of age and more than 30% of the population over 60 years of age suffer from PFOA [1,6]. These comprehensive mechanisms of OA development are highly relevant to the patellofemoral joint, where biomechanical forces are particularly concentrated [3,4]. Alteration of the anatomy or congruence of the patellofemoral joint, whether congenital or acquired (e.g., trochlear dysplasia, patella alta, lateralization of the tibial tuberosity, and subluxation of the patella), leads to abnormal patellar alignment and tracking abnormalities. Such tracking abnormalities result in an irregular and abnormal distribution of mechanical stress across the joint surface, initiating localized cartilage degradation, subchondral sclerosis, and synovial inflammation characteristic of the whole-joint OA disease process [3,4,7,8]. Magnetic resonance imaging (MRI) signs of patellar dislocation include patellofemoral ligament disruption, patellar contusion, cartilaginous defects, and joint effusion [9,10]. The tibial tuberosity–trochlear groove (TT-TG) distance has also been used to quantify tibial tuberosity lateralization [9,11]. However, the TT-TG distance reflects only one of the anatomically relevant factors that lead to patellar instability and cannot be used to assess patellar lateralization without tibial tuberosity abnormality [9]. We have reported that the distance of patellar lateralization in a coronal superimposed MR image (coronal MRI superimposition technique) can be a useful method for evaluating patellar dislocation and have suggested a 7 mm threshold for the best diagnostic discrimination [9]. Although our previous study focused on the assessment of patellar dislocation, the novelty of the current study is to evaluate the relationship between this morphological parameter and the presence and severity of patellofemoral osteoarthritis (PFOA), using an entirely independent patient group.

In this study, we correlated the presence of PFOA with the TT-TG distance and the distance of patellar lateralization in the same knee. We also evaluated the correlation between patellar lateralization and PFOA severity.

## 2. Materials and Methods

### 2.1. Case Selection

**Figure 1:**
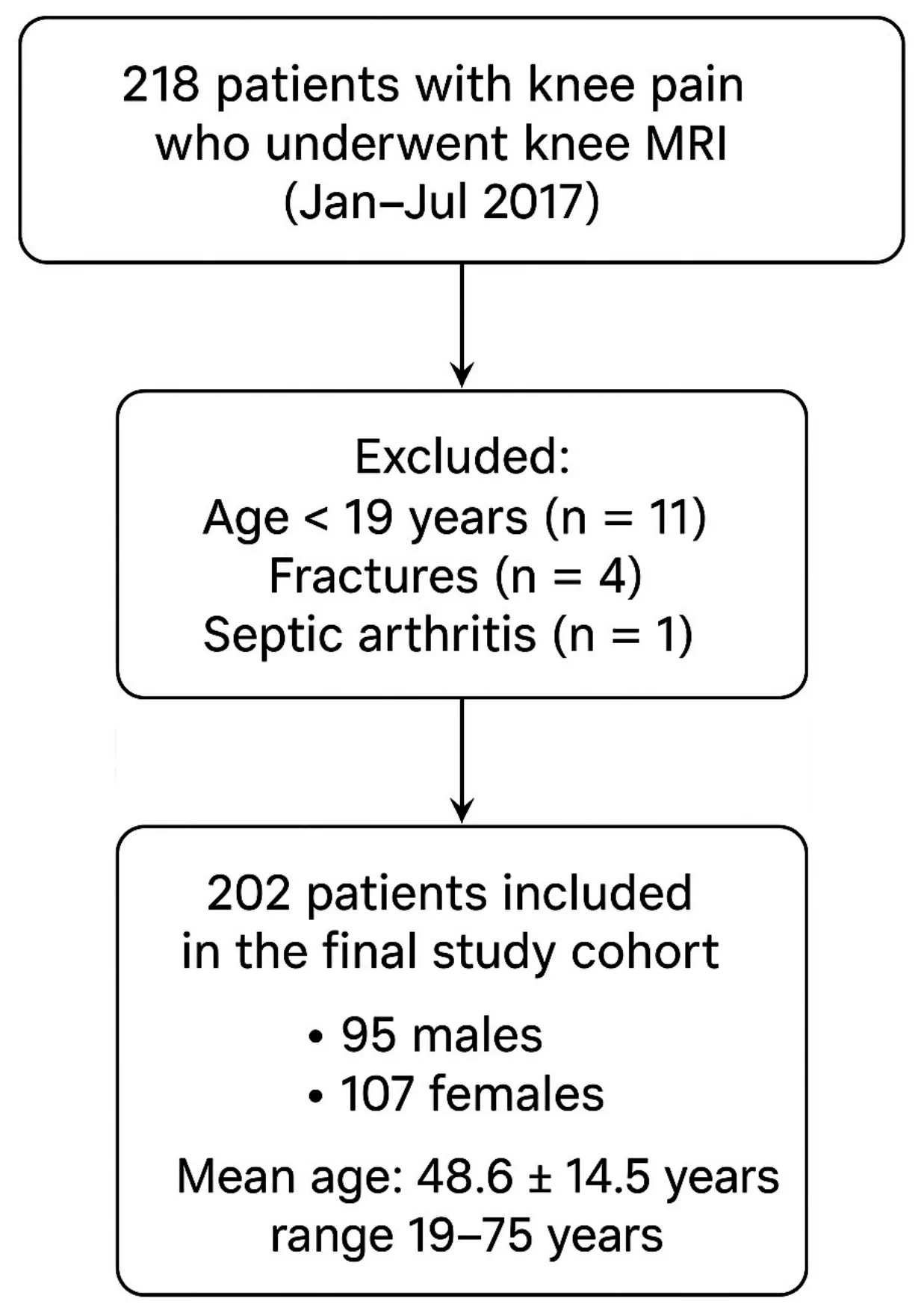
Flow chart of enrollment.

The Kangbuk Samsung Hospital Institutional Review Board (KBSMC 2019-11-053) approved our study and waived the requirement for informed consent due to the retrospective design of the study. We retrospectively evaluated 218 consecutive patients who had knee pain and underwent knee MRI between January 2017 and July 2017. Because all participants were selected from these symptomatic patients, it should be explicitly noted that the control group in our study consisted of patients presenting with knee pain, rather than asymptomatic healthy volunteers. Among these patients, we excluded those who were under 19 years of age (eleven cases), had septic arthritis (one case), or had a history of previous knee surgery and distinct trauma, including fractures (four cases). In all included cases, only the right knee was evaluated. The study group consisted of 95 males and 107 females (average age: 48.6 ± 14.5 years; range: 19 to 75 years; Figure 1).

### 2.2. MRI Parameters

All magnetic resonance (MR) examinations were performed according to the same protocol using a 3.0 T MRI scanner (Achieva, Philips, Best, the Netherlands) and an eight-element (SENSE) phased-array knee joint-dedicated coil with the patient’s knee in extension. To control limb rotation and maintain a neutral alignment, the patient’s foot and ankle were positioned neutrally and carefully secured within the coil. Sequence and imaging parameters are summarized in Table 1.

### 2.3. Measure of the Patellar Lateralization and TT-TG Distance

**Figure 2:**
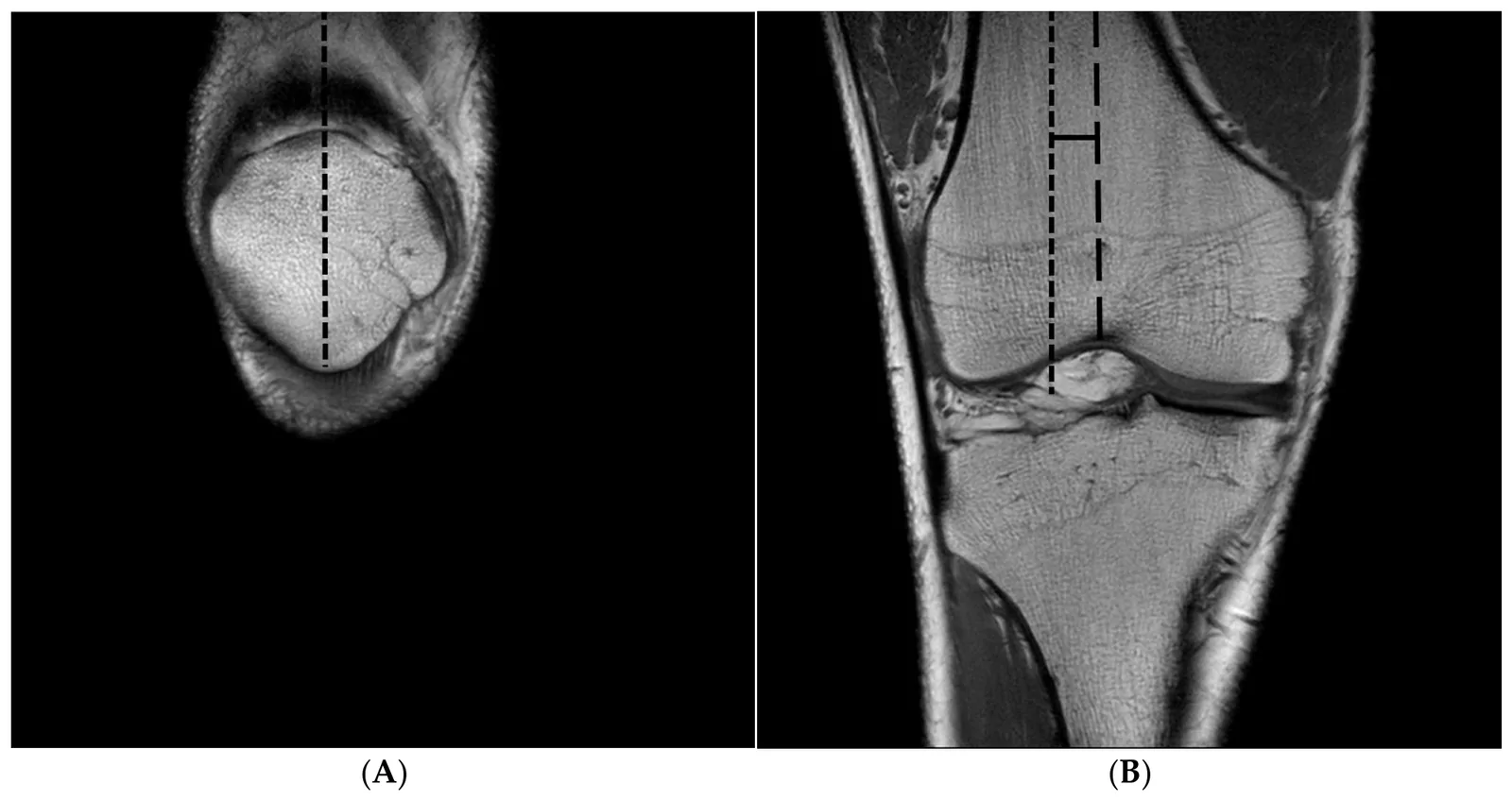
Measurement of the patellar lateralization using the coronal MRI superimposition technique on a coronal T1-weighted image (TR/TE, 709/10). (**A**) A vertical line is drawn and then copied from the lowest tip of the patella (short dotted line). (**B**) The distance of patellar lateralization (solid line) was measured between the pasted patellar long axis (short dotted line) and the deepest portion of the trochlear notch (long dotted line).

All image analyses and line transfers were performed using a picture archiving and communication system (INFINITT PACS, version 7.0). In order to measure the MRI-defined lateral patellar displacement, we used a coronal MRI superimposition technique. Because the coronal MRI superimposition technique relies on basic PACS tools, it is highly efficient and takes approximately 1 min per case to perform. First, we measured the distance between the long axis of the patella and the deepest portion of the trochlear notch. For slice selection and the line transfer method, we utilized the reference line copy-and-paste tool provided by the PACS software, version 7.0. Specifically, we identified the specific coronal T1-weighted slice demonstrating the lowest tip of the patella. We drew a vertical line from the lowest tip of the patella in the T1-weighted image of the coronal plane (Figure 2A, short dotted line), copied the annotated line, and pasted it to the plane that had the deepest portion of the intercondylar fossa in the same image sequence as the coronal plane (Figure 2B). Then, we drew another vertical line from the deepest portion of the intercondylar fossa parallel to the first line (Figure 2B, long dotted line). The distance between the second and third lines indicates the degree of patellar lateralization (Figure 2B, solid line).

**Figure 3:**
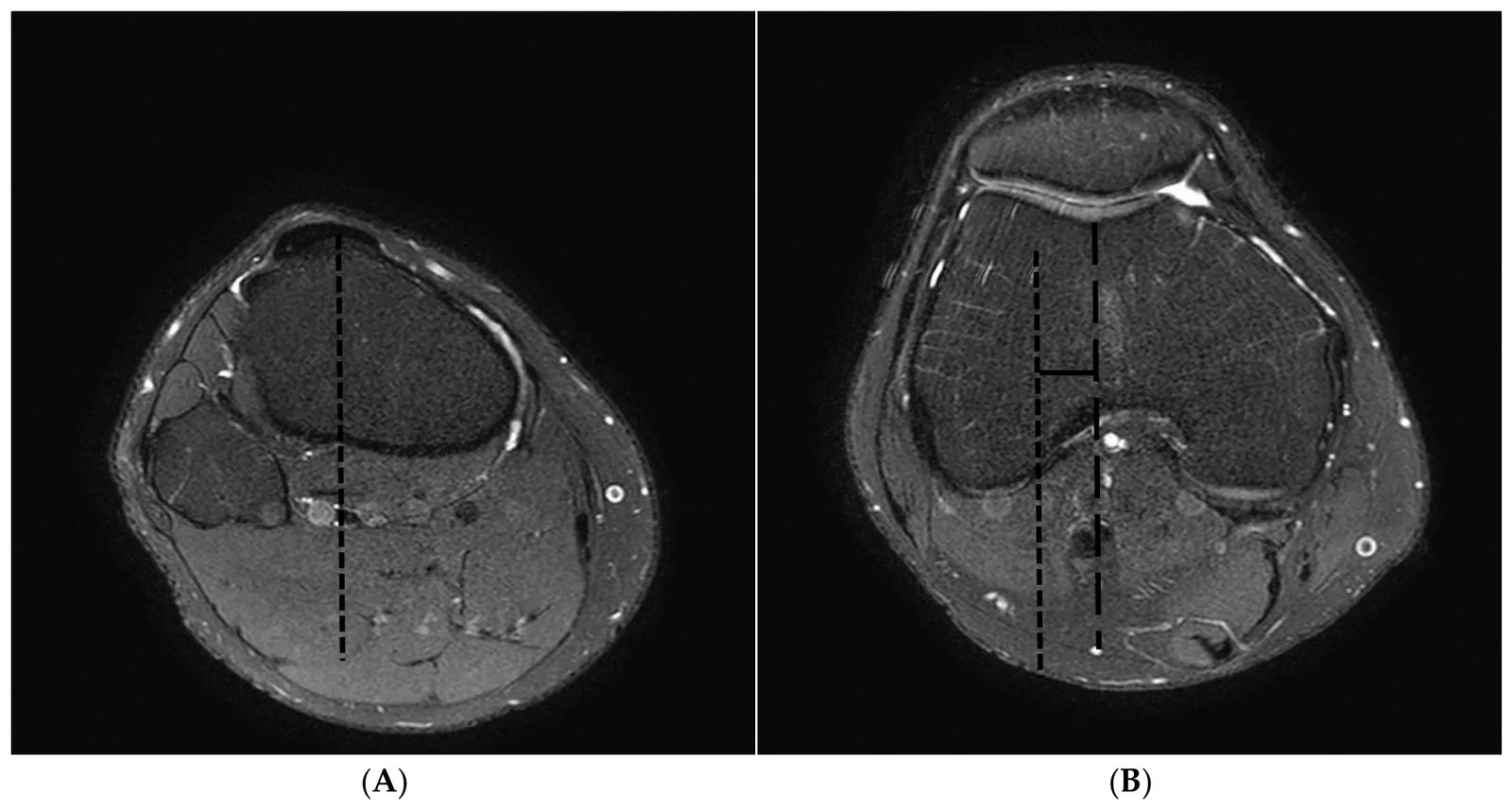
Measurement of the TT-TG distance (tibial tuberosity to the trochlear groove) on an axial T2-weighted image (TR/TE, 4632/70). (**A**) The vertical line from the most anterior portion of the tibial tuberosity (short dotted line) is drawn and then copied. (**B**) The TT-TG distance (solid line) was measured between the vertical line pasted at the most anterior portion (short dotted line) and the deepest portion of the trochlear groove (long dotted line).

In order to measure the TT-TG distance, a perpendicular line was drawn at the most anterior portion of the tibial tuberosity notch in the fat-saturated T2-weighted image of the axial plane (Figure 3A, short dotted line). The line was copied and pasted to the image of the deepest point of the trochlear groove in the same image sequence (Figure 3B). Then, a third line was drawn parallel to the second line at the deepest point of the trochlear groove (Figure 3B, long dotted line). The distance between the second and third lines is the TT-TG distance (Figure 3, solid line).

### 2.4. Image Analysis of the PFOA

**Figure 4:**
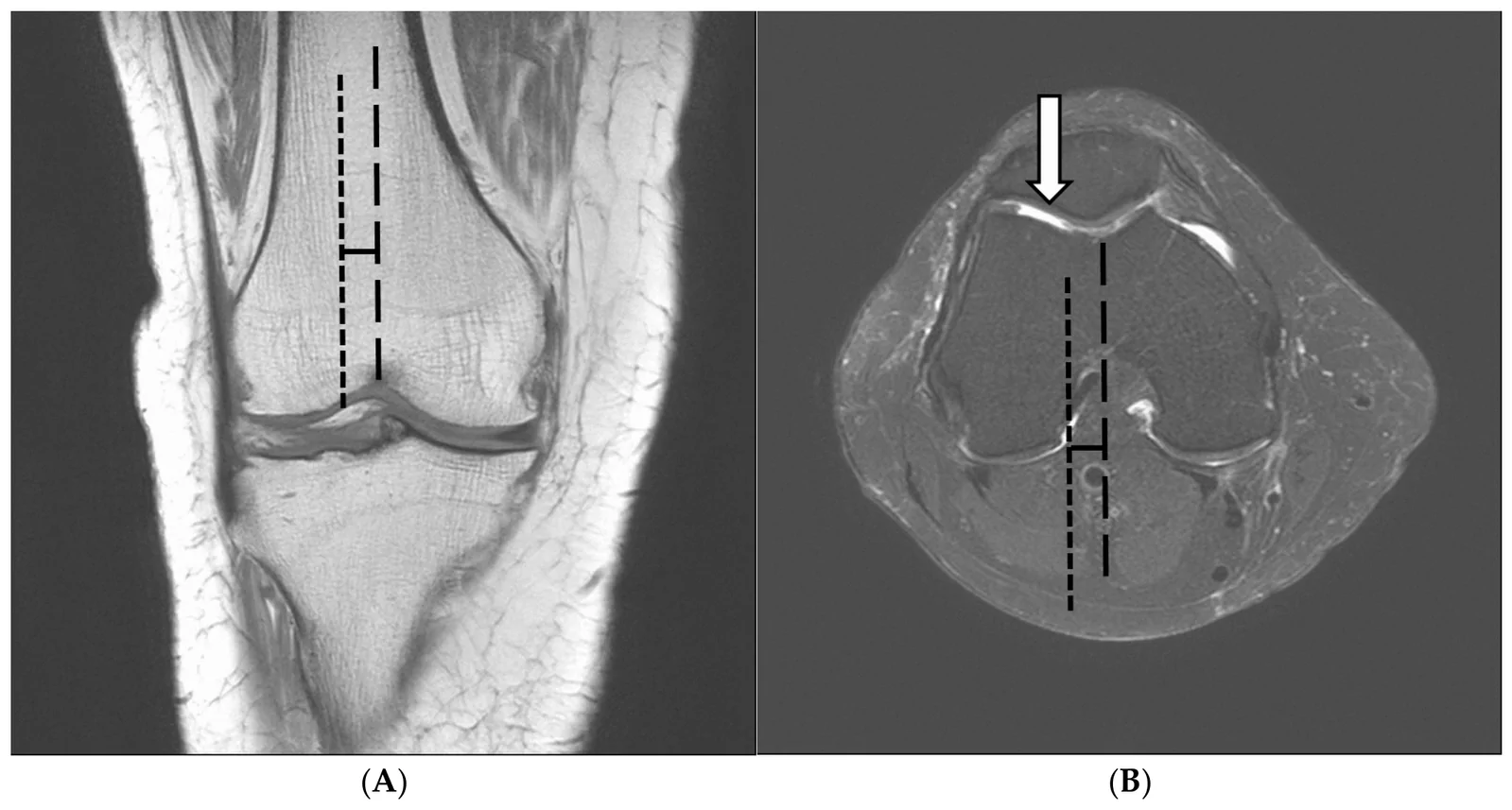
A patient with knee pain. (**A**) The patellar lateralization distance on a coronal MRI (TR/TE, 709/10) is 7.16 mm, as measured between the short dotted and long dotted lines. (**B**) The TT-TG distance on an axial MRI (TR/TE 4632/70) is 8.13 mm. The full-thickness cartilage defect in a patellofemoral joint with bone exposure (arrow) is classified as ICRS Grade 4. Solid line is distance, dotted line is tibial tuberosity and long dotted line is trochlear groove.

All 202 MRI scans were retrospectively and independently evaluated in a randomized order by two musculoskeletal radiologists (with 3 and 15 years of experience). The raters were completely blinded to the patients’ clinical information, including symptoms, prior radiographs, the results of the measurements (such as patellar lateralization and TT-TG distance), and each other’s initial assessments. We assessed patellofemoral joint osteoarthritic lesions based on typical imaging findings such as signal change and cartilage defects [1]. We assessed the medial and lateral femoral condyle and patella of the patellofemoral joint for cartilage lesions [12]. Assessment of the severity of cartilage lesions was based on the International Cartilage Repair Society (ICRS) classification system (Table 2) (Figure 4B) [1,13]. Two readers assessed cartilage lesions such as signal change and cartilage defects on the MR images independently. Any discrepancies between the two raters were resolved by consensus.

### 2.5. Statistical Analysis

We first categorized descriptive statistics for all variables used in the study (TT-TG distance, patellar lateralization, and OA grades). We used the intra-class correlation coefficient (ICC, two-way random-effects model with absolute agreement) to quantify agreement between both readers. Interpretation of ICC values was as follows: >0.75, excellent agreement; 0.40–0.75, fair-to-good agreement; <0.40, poor agreement [14]. After dividing the study group into a patellar lateralization group and a control group according to the distance between the patellar long axis and the deepest portion of the trochlear notch using a 7 mm threshold, the association between patellar lateralization and PFOA severity was analyzed [9].

As we hypothesized that patellar lateralization may increase the prevalence of advanced PFOA (ICRS Grades 2–4), we tested this trend using the Cochran–Armitage test [15,16]. After dividing the study group into a PFOA group (ICRS Grades 2–4) and a non-PFOA group (ICRS Grades 0–1) according to the interpretation of each reader, we compared the distance of the patellar lateralization using Student’s *t*-test. To compare the TT-TG distance between the non-PFOA group and the PFOA group, we also used Student’s *t*-test. We also compared the distance of patellar lateralization between the two groups in the same manner.

Next, we divided the study group into a severe PFOA group (ICRS Grades 2–4) and a non-severe PFOA group (ICRS Grades 0–1). We compared the TT-TG distance and the distance of patellar lateralization in the same manner. Before conducting the independent *t*-tests, the assumption of normality was evaluated, and the homogeneity of variances was confirmed using Levene’s test. To account for multiple comparisons, appropriate adjustments (e.g., the Bonferroni correction) were applied to the significance level to control the Type I error rate. As this was a retrospective study, an a priori sample size calculation was not performed. Furthermore, to provide a more comprehensive evaluation of the clinical significance, effect sizes (Cohen’s d) and 95% confidence intervals (CIs) for the mean differences were calculated and reported alongside the *p*-values.

Statistical analyses were performed using version 18.0 of the statistical software PASW (IBM, Armonk, NY, USA). If the calculated *p*-value was below 0.05, the result was considered to be statistically significant.

## 3. Results

The interobserver agreement was excellent for both continuous measurements. Specifically, the ICC for the TT-TG distance was 0.980 (95% CI, 0.973–0.985), and the ICC for patellar lateralization was 0.984 (95% CI, 0.978–0.988). To further assess measurement reliability, we calculated the standard error of measurement (SEM) and the minimum detectable change (MDC). The SEM was 0.45 mm for the TT-TG distance and 0.38 mm for patellar lateralization. The corresponding MDCs were 1.25 mm and 1.05 mm, respectively, indicating excellent measurement precision relative to the observed group differences. The mean distance of the patellar lateralization in the PFOA group was greater than that of the control group (*p* = 0.011 and 0.001, Table 3); however, there was no significant difference in the TT-TG distance between the two groups. Also, we noted that as the ICRS grade of the PFOA rose, the proportion of patellar lateralization increased more than normal in each grade (*p* = 0.009, Table 4). There were statistically significant differences in the TT-TG distance and patellar lateralization between the non-severe group (ICRS Grades 2–4) and the severe PFOA group (ICRS Grades 2–4) for both interpreters (*p* = 0.029 and 0.014 for TT-TG distance; *p* = 0.005 and 0.002 for patellar lateralization; Table 5).

In multivariable logistic regression analyses adjusted for age and sex, both TT-TG distance and patellar lateralization remained significantly associated with severe PFOA. For Reader 1, the adjusted ORs per 1 mm increase were 1.128 (95% CI, 1.009–1.261; *p* = 0.034) for TT-TG distance and 1.245 (95% CI, 1.083–1.432; *p* = 0.002) for patellar lateralization. Similar results were observed for Reader 2, with adjusted ORs of 1.125 (95% CI, 1.004–1.260; *p* = 0.042) for TT-TG distance and 1.234 (95% CI, 1.077–1.414; *p* = 0.002) for patellar lateralization (Table 6).

## 4. Discussion

A skyline view of the patella through plain radiography can provide information on the anatomy of the patellofemoral joint [9,17]. The skyline view is performed with a knee flexion angle of 45°, which can cause the displaced patella to return to a nondisplaced position, leading to a false-negative result [18,19]. However, knee MRI is performed with the knee extended, which allows for an accurate evaluation of patellar lateralization [9]. Vertical lines at the lowest portion of the patella and the deepest portion of the trochlear notch were used as reference lines. The patella is a small, firm, triangular bone located in front of the knee joint, with its tip facing distally and being the origin of the patellar ligament; thus, the lower tip of the patella can serve as a reliable landmark [9,20]. Although the 7 mm threshold for patellar lateralization was originally established based on a previous study of patellar instability [9], our results demonstrate that this cutoff value also effectively reflects the anatomical severity associated with PFOA.

In this study, we found that patellar lateralization in the PFOA group was greater than that in the normal group; however, there was no difference in the TT-TG distance between groups. Tsavalas et al. [1] reported a similar result, with no significant difference in the TT-TG distance between the PFOA and control groups. Noehren et al. reported a markedly different result: the TT-TG distance in the PFOA group was significantly greater than that in the control group [21]. However, in that study, only cases with ICRS Grades 3 and 4 were included in the disease group, and the mean age of their study group was 32 years, whereas our mean age was 49 years. When we classified the study group into a non-severe group (ICRS Grade 0 and Grade 1) and a severe PFOA group (ICRS Grades 2–4), we found similar results (Table 5) [21]. The TT-TG distance reflects only one of the anatomical factors responsible for patellar lateralization subsequent to OA and cannot be used to evaluate patellar lateralization without tibial tuberosity abnormalities [9]. By using the distance of patellar lateralization on coronal MRIs with the coronal MRI superimposition technique, we were able to effectively evaluate its association with PFOA. Patellar dislocation represents the dissociation of the articulating surface between the patella and femur. Acquired alterations in the anatomy of the patellofemoral joint (PFJ) may contribute to patellar dislocation, which is associated with PFJ cartilage lesions and subsequent OA [1,7,8,21]. Many studies have reported that patellar dislocation is associated with PFOA [21,22,23,24,25]. Patellar dislocation is highly associated with PFOA [8,26]. Similar to previous studies, we also found that the higher the ICRS grade of PFOA, the higher the proportion of patients with patellar lateralization (when patellar MRI-defined lateral patellar displacement is defined by the distance of lateralization on coronal MRIs using a 7 mm threshold). This indicates that patients with patellar lateralization are strongly associated with advanced PFOA. This may also indicate that patients with advanced PFOA have an increased risk of disruption of the medial ligamentous stabilizer, as previously reported [9].

When we reclassified the group into severe and non-severe PFOA, there was a significant difference in TT-TG distance and patellar lateralization. One study group identified a different predominant factor affecting PFOA progression in the study. They found that the TT-TG distance was likely to be greater in patients with advanced PFOA than in those with early PFOA [27,28]. Because the TT-TG distance is regarded as the patellar force vector, the rotational abnormality may be correlated with the severity of PFOA. Despite this speculation, the TT-TG distances measured in both groups were within the normal range reported in the literature, similar to our findings [11].

Furthermore, while our findings demonstrate a strong correlation, we also consider the possibility of a reverse association. It is highly reasonable to consider that patellar lateralization may result from the degeneration of the patellofemoral joint rather than being the primary cause. As osteoarthritis progresses, cartilage loss, subchondral bone alteration, and subsequent ligamentous laxity can significantly alter joint biomechanics, potentially leading to secondary lateralization of the patella. This secondary lateralization may be further aggravated by the other pre-existing anatomical abnormalities, such as trochlear dysplasia or patella alta. Due to the retrospective design of our study, we cannot definitively determine the exact cause-and-effect relationship.

Our study had some limitations. First, this was a retrospective study. Second, although age and sex were included as covariates in the multivariable analyses, other potential confounding factors, such as BMI, patella alta, trochlear dysplasia, lower-extremity alignment, and concomitant tibiofemoral osteoarthritis, were not systematically assessed and could not be incorporated into the models [29,30,31,32]. Therefore, residual confounding cannot be excluded, and the observed associations should not be interpreted as causal. Future prospective studies incorporating these factors are warranted. Third, we excluded cases under 20 years of age, but there was no upper limit of age. This may have affected the results, as older patients may have many factors associated with osteoarthritis. Fourth, there is a limitation regarding the MRI protocol used for cartilage assessment. We evaluated the cartilage using standard routine MRI with a 3 mm slice thickness. This standard slice thickness may limit the detection of very small, focal, or early-stage (e.g., ICRS Grade 1) cartilage lesions, which could potentially lead to an underestimation of early cartilage degeneration compared to thinner slices or 3D high-resolution cartilage-specific sequences. Fifth, our measurements were based on static MRI acquired with the knee in full extension. Static MRI assessment has inherent limitations in evaluating dynamic patellar tracking. Patellar instability and lateralization often manifest during active knee flexion and extension. Therefore, static images may not fully reflect the dynamic biomechanical abnormalities present during functional joint movement. Sixth, the patient group was collected in 2017. However, because our study evaluates anatomical and morphological relationships between patellar lateralization and PFOA, the age of the dataset does not alter the biological validity of our findings. Furthermore, the 3.0 T MRI scanner and imaging protocols used in this study are widely used in clinical settings, so they would be highly applicable. Seventh, while we reported excellent inter-reader reliability (ICC) for the measurements (patellar lateralization and TT-TG distance), we did not calculate the inter-reader agreement for the ICRS grading. Instead, any discrepancies were resolved by consensus. Nevertheless, our study had certain strengths. We evaluated patellar lateralization using a coronal MRI superimposition technique, which provides a reliable anatomical assessment, and showed that patellar lateralization measured using the coronal MRI superimposition technique was significantly correlated with PFOA and its severity, serving as a valuable complementary parameter alongside the TT-TG distance [9]. Therefore, the coronal MRI superimposition technique can be used in clinical settings. By extension, it would be helpful to determine the mechanism of PFOA development and the predisposing factors, particularly for patients with PFOA within the normal range of TT-TG distance. Further prospective studies are required to elucidate its role and mechanism.

## 5. Conclusions

In conclusion, the measurement of patellar lateralization using the coronal MRI superimposition technique on MRI is significantly associated with PFOA and its severity, although its potential prognostic value requires further prospective and independent validation studies. Thus, the measurement of patellar lateralization using the coronal MRI superimposition technique would be a useful method for evaluating the relationship between patellar lateralization and PFOA.

## Figures and Tables

**Table 1:** Routine MR sequences and parameters.

Imaging Parameter	Coronal T1 ^1^ FSE	Coronal ^2^ FS T2 FSE	Sagittal FS ^3^ PD FSE	Sagittal T2 FSE	Axial FS T2 FSE
TR (msec)	550–750	3800–9800	3100–5000	2900–4500	4400–7500
TE (msec)	10–15	65–75	30–36	72–100	65–80
Flip angle (°)	90	90	90	90	90
Matrix size (pixel)	256 × 346	352 × 224	320 × 240	356 × 280	320 × 238
Field of view (mm)	160	160	160	160	160
Slice thickness (mm)	3	3	3	3	3
Inter-slice gap (mm)	0.3	0.3	0.3	0.3	0.3
Bandwidth (kHz)	270	270	256	240	295
Echo train length	6	16	12	15	16
Signal average	1	1	1	1	1
Scan time (min:s)	2:50	3:00	2:30	2:40	2:30

^1^ FSE = fast spin-echo; ^2^ FS = fat saturation; ^3^ PD = proton density.

**Table 2:** International Cartilage Repair Society (ICRS) classification system of cartilage lesions.

Grade	Cartilage Morphology on MRI
0	Normal
1	Abnormal increased signal with intact articular surface
2	Partial-thickness defect < 50%
3	Partial-thickness defect 50–99%
4	Full-thickness defect with exposure of subchondral bone and marrow edema

**Table 3:** Comparison of mean TT-TG distance and patellar lateralization between non-PFOA and PFOA groups.

		Non-PFOA (*n* = 155)	PFOA (*n* = 47)	95% CI	*p*-Value	Cohen’s d
Reader 1	TT-TG distance	7.21 (±2.95, 4.26–10.16)	7.98 (±3.44, 4.54–11.42)	[−1.659, 0.121]	0.090	0.24
	Patellar lateralization	4.74 (±2.81, 1.93–7.55)	5.82 (±3.12, 2.7–8.94)	[−1.912, 0.254]	0.011	0.37
Reader 2	TT-TG distance	7.36 (±3.06, 4.3–10.42)	8.08 (±3.21, 4.87–11.29)	[−1.611, 0.164]	0.110	0.41
	Patellar lateralization	4.78 (±2.82, 1.96–7.6)	6.17 (±3.15, 3.02–9.32)	[−2.234, −0.557]	0.001	0.20

**Table 4:** Correlation of patellar lateralization and patellofemoral osteoarthritis severity.

Interpreter	Group	Grades of the International Cartilage Repair Society						*p*-Value	Effect Size (Cramer’s V)
		0	I	II	III	IV	Total		
Reader 1	No lateralization (%)	96 (61.9)	14 (9.0)	19 (12.3)	11 (7.1)	15 (9.7)	155 (100)	0.009	0.19
	Patellar lateralization (%)	21 (44.7)	4 (8.5)	7 (14.9)	4 (8.5)	11 (23.4)	47 (100)		
	Total	117	18	26	15	26	202		
Reader 2	No lateralization (%)	98 (65.8)	8 (5.4)	18 (12.1)	13 (8.7)	12 (8.1)	149 (100)	0.009	0.22
	Patellar lateralization (%)	25 (47.2)	4 (7.5)	6 (11.3)	6 (11.3)	12 (22.6)	53 (100)		
	Total	123	12	24	19	24	202		

**Table 5:** Association of TT-TG distance and patellar lateralization between non-severe PFOA and severe PFOA (ICRS Grades 2–4).

		<PFOA Grade 2 (*n* = 135)	≥PFOA Grade 2 (*n* = 67)	95% CI	*p*-Value	Cohen’s d
Reader 1	TT-TG distance	7.20 (±3.04)	8.23 (±3.37)	[−1.962, −0.104]	0.029	0.33
	Patellar lateralization	4.78 (±2.79)	6.02 (±3.23)	[−2.110, −0.376]	0.005	0.42
Reader 2	TT-TG distance	7.27 (±3.05)	8.41 (±3.18)	[−2.051, −0.228]	0.014	0.37
	Patellar lateralization	4.87 (±2.81)	6.23 (±3.25)	[−2.232, −0.488]	0.002	0.46

**Table 6:** Age- and sex-adjusted associations of TT-TG distance and patellar lateralization with severe patellofemoral osteoarthritis.

Parameter	Reader	Adjusted OR *	95% CI	*p* Value
TT-TG distance	1	1.128	1.009–1.261	0.034
TT-TG distance	2	1.125	1.004–1.260	0.042
Patellar lateralization	1	1.245	1.083–1.432	0.002
Patellar lateralization	2	1.234	1.077–1.414	0.002

*Notes:* Adjusted for age and sex. Severe PFOA was defined as ICRS grades 2–4. ORs are reported per 1 mm increase in TT-TG distance or patellar lateralization. * odd ratio.

## Data Availability

No new data were created or analyzed in this study. Data sharing is not applicable to this article.
